# Functional Characterization of a Ketoreductase-Encoding Gene *med*-ORF12 Involved in the Formation of a Stereospecific Pyran Ring during the Biosynthesis of an Antitumor Antibiotic Medermycin

**DOI:** 10.1371/journal.pone.0132431

**Published:** 2015-07-10

**Authors:** Qiang He, Le Li, Tingting Yang, Ruijuan Li, Aiying Li

**Affiliations:** 1 State Key Laboratory of Microbial Technology, School of Life Sciences, Shandong University-Helmholtz Institute of Biotechnology, Shandong University, Jinan, China; 2 School of Life Sciences, Central China Normal University, Wuhan, China; University of Strathclyde, UNITED KINGDOM

## Abstract

Medermycin, a polyketide antibiotic, possesses strong bioactivity against a variety of tumors through a novel mechanism and is structurally featured with a pyran ring containing two chiral centers (3*S *and 15*R*). By far the biosynthetic origin of such enantiomerical conformations still remains obscure. In the present study, we reported the functional characterization of a proposed ketoreductase Med-ORF12 encoded by medermycin biosynthetic cluster and revealed its involvement in the stereochemical control at C3 center of medermycin. Firstly, bioinformatics analysis of Med-ORF12 suggested that it belongs to a group of stereospecific ketoreductases. Next, a Med-ORF12-deficient mutant was obtained and LC/MS measurements demonstrated that medermycin production was completely abolished in this mutant. Meanwhile, it was found that two shunt products were accumulated at the absence of Med-ORF12. Finally, the reintroduction of Med-ORF12 into this mutant could restore the production of medermycin. In a conclusion, these data supported that Med-ORF12 is essential for the biosynthesis of medermycin and performs its role as a stereospecifc ketoreductase in the tailoring steps of medermycin biosynthetic pathway.

## Introduction

Streptomycetes are great sources of abundant natural products with potential pharmaceutical and agricultural applications, including antibiotics, immune-modulators, antitumor agents and so on [1–2]. Among these natural products, aromatic polyketides (PKs) are valued impressively in human medicine and agriculture, due to their highly structural and bioactive diversity.

**Fig 1:**
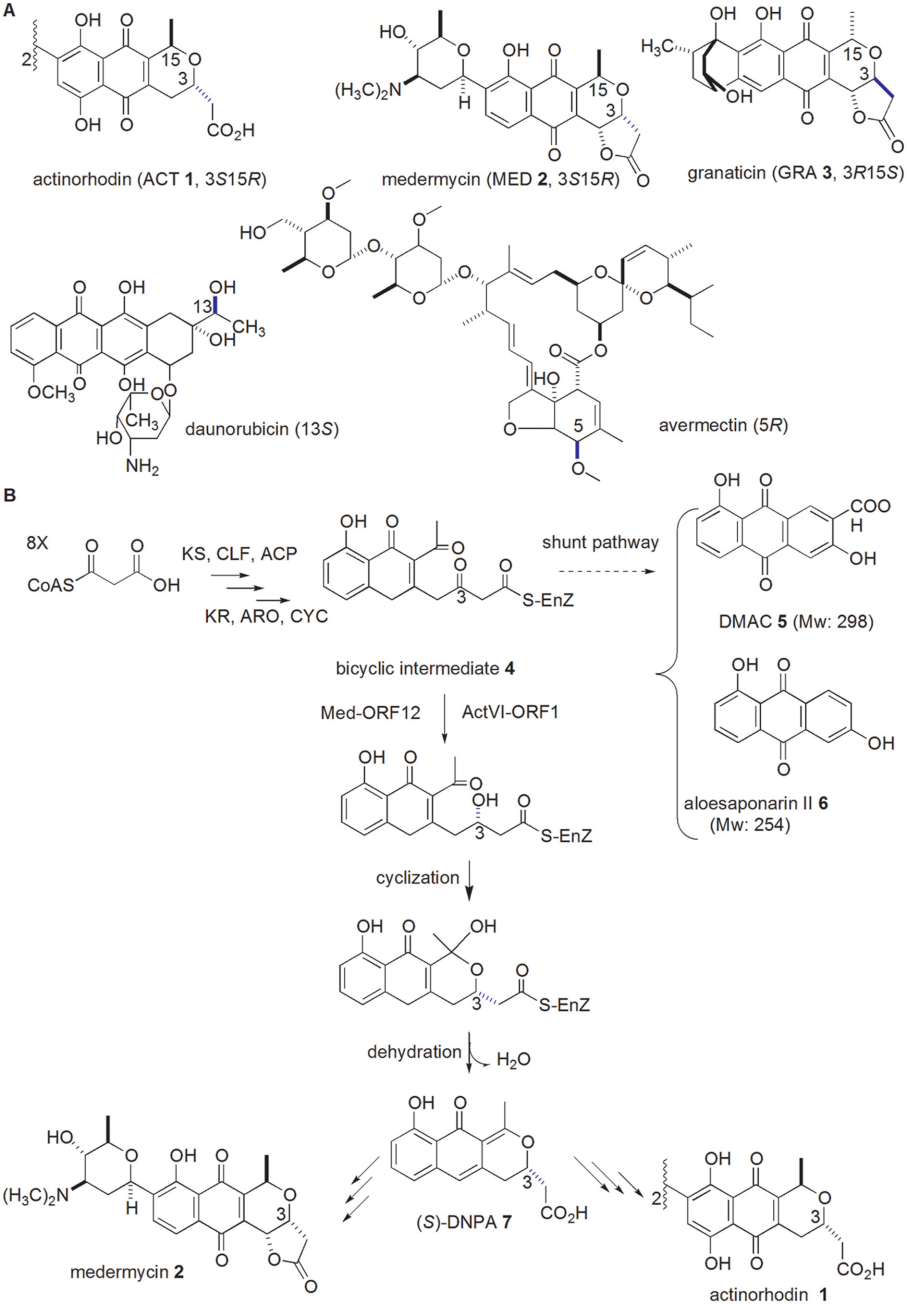
Examples of polyketide antibiotics with stereospecific centers and proposed biosynthetic pathways of medermycin and actinorhodin. A: three members of BIQ family shares opposite conFig uration at C3 and C15 (either 3*S*15*R*, or 3*R*15*S*). Two well-known antibiotics, daunorubicin and avermectin, have more than one stereospecific center and genes for stereochemical control at C13 and C5 for these two antibiotics respectively will be discussed in this study. B: Biosynthetic pathways of MED **2** and ACT **1** were proposed to share common earlier stages to produce a bicyclic intermediate **4**. The formation of this intermediate was catalyzed by polyketide synthases (minimal PKS: KS, CLF and ACP) and related enzymes (KR, ARO and CYC). It could be converted into two shunt products (DMAC **5** and aloesaponarin II **6**) in the *act*VI-ORF1-deficient mutant strain. The keto-reduction at C-3 of this bicyclic intermediate **4** in ACT **1** pathway was performed by ActVI-ORF1, probably by Med-ORF12 in MED **2** pathway, followed by spontaneous cyclization and dehydration, leading to the production of DNPA **7** with 3*S* configuration.

Over thirty years, taking actinorhodin (ACT, **1**) as a model in the family of benzoisochromanequinones (BIQs, also referenced as pyranonaphthoquinones) [3–5] (Fig 1A), the biosynthesis of aromatic polyketide antibiotics has been extensively studied, especially at the earlier stages: A multifunctional enzyme complex (namely minimal polyketide synthases (minimal PKS, composed of ketosynthase (KS), chain length factor (CLF) and acyl carrier protein (ACP)) is responsible for the formation of the polyketide chain with a certain length, followed by ketoreduction, aromazation and cyclization catalyzed by ketoreductase (KR), aromatase (ARA) and cyclase (CYC) in a serial of reactions, leading to the formation of aromatic polyketide skeletons [6]. Subsequent tailoring modification steps, including methylation, dimerization, oxidation, glycosylation and so on, result in the accumulation of final products with a high diversity in their structures and bioactivity [7]. Compared to the earlier stages, most of these tailoring modification steps still have yet to be investigated, even for the biosynthetic pathway of well-studied ACT **1** [4].

Medermycin (MED, **2**), another member in the BIQ family [3], possesses strong bioactivity against many types of tumor cells through a novel mechanism, besides its inhibitory activity against Gram positive bacteria [8–10]. It is featured with a fused three-ring structure composed of a benzene ring, a quinone and a stereospecific pyran ring.

Stereochemical control is a distinguished characteristic in BIQ family, such as 3*S*15*R*-conFig uration for ACT **1** and MED **2** and 3*R*15*S*-conFig uration for granaticin (GRA **3**) (Fig 1A) [3–5]. Stereochemical control is also a common and important tailoring modification during the biosynthetic pathways of many other polyketide antibiotics, including well-known antitumor agent daunorubicin (Fig 1A) [11]. Generally, the stereochemistry of a functional group influences remarkably on the bioactivity of natural products [12–13], hence the biosynthetic problem concerning the formation of this pyran ring in MED **2** has been an important issue [12].

**Fig 2:**
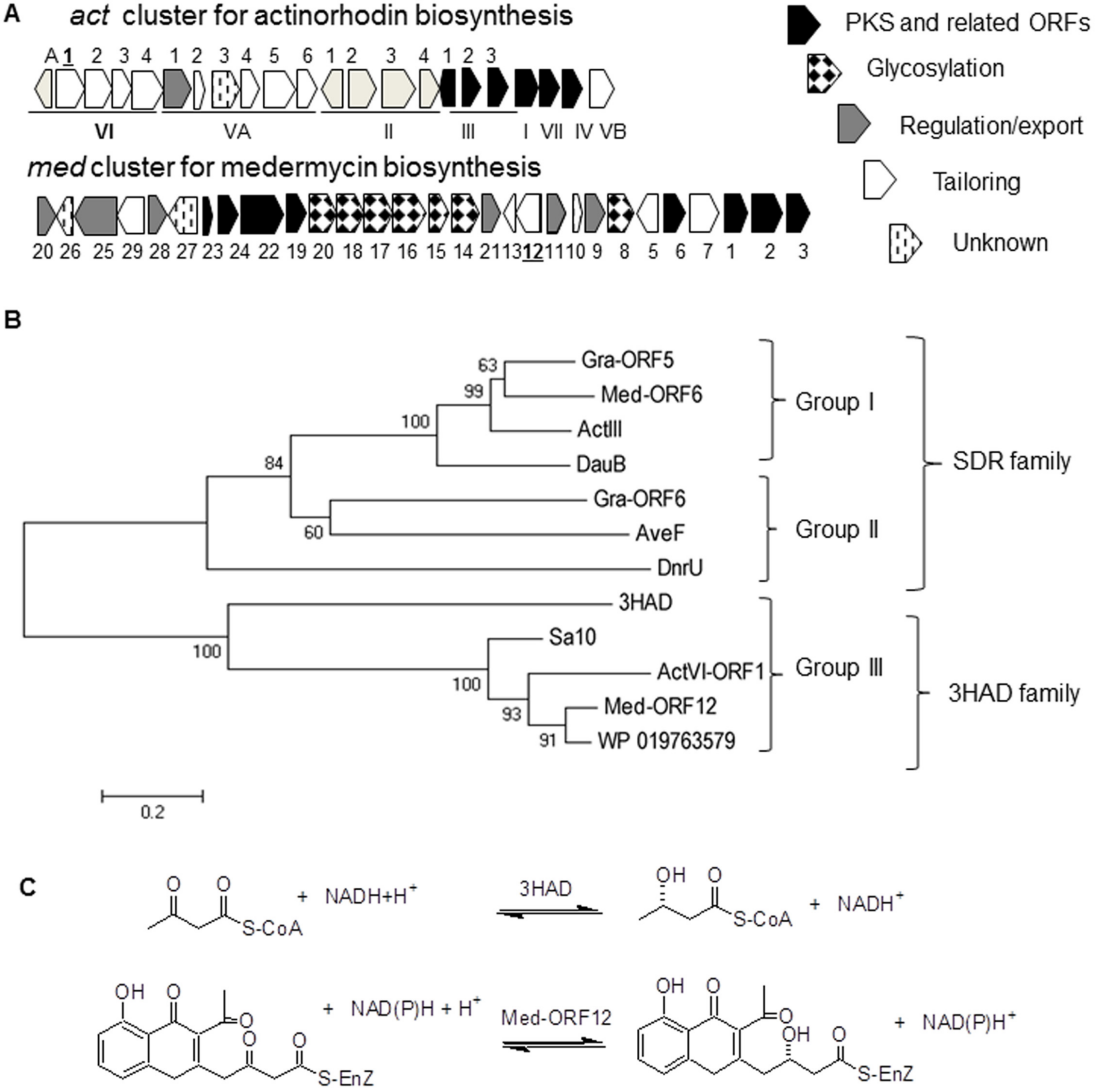
Comparison between two biosynthetic gene clusters for medermycin and actinorhodin and phylogenetic analysis of Med-ORF12 and its homologies. A: Predicted function of genes in the clusters for ACT **1** and MED **2** respectively is indicated as bars with different filling styles. B: Phylogenetic tree was established using the amino acid sequences of Med-ORF12 and Med-ORF6 (accession number: BAC79036 and BAC79042, for MED **2**) from *Streptomyces sp* AM-7161, ActVI-ORF1 and ActIII (NP_629223 and NP_629236, for ACT **1**) from *S*. *coelicolor* A3(2), DauB (AAA87616 for aklaviketone) from *Streptomyces sp*., Gra-ORF5 and Gra-ORF6 (P16542 and P16543 for GRA **3**) from *Streptomyces violaceoruber*, AveF (NP_822111 predicated as ketoreductase for C5 of avermectin) from *Streptomyces avermitilis*, DnrU(Q_9ZAU1, marked as daunorubicin C-13 ketoreductase) from *Streptomyces peucetius*, Sa10 (ACK77759 for indigoidine/auricin) from *Streptomyces aureofaciens*, 3HAD (3HAD_B) from human heart and a hypothetical protein (WP_019763579) from *Streptomyces sp* Wigar10. The bar indicated the evolutionary distance. The numbers on branch nodes were percentages of 1000 sets of bootstrap supports. 3HAD: 3-hydroxyacyl-CoA dehydrogenase protein family; SDR: short-chain alcohol dehydrogenases family. C: Comparison between the reactions catalyzed by 3HAD and Med-ORF12 respectively.

The 29-gene-containing cluster for the biosynthesis of MED **2** was cloned and sequenced in 2003 [14]. Comparative studies between *med* and *act* gene clusters proposed that the biosynthetic pathways of ACT **1** and MED **2** share similar earlier stages to produce a bicyclic intermediate **4** (Figs 1B and 2A). Many homologous genes are present on both of these two gene clusters, some of which are proposed for post-PKS modification of ACT **1** and MED **2**. For example, *act*VI-ORF1 and *med*-ORF12 share 65/57% of similarity/identity (Fig 2A) [14], implying they share a common role in the pathways of these two compounds.

Evidences *in vitro* and *in vivo* proved that *act*VI-ORF1 encodes a stereospecific ketoreductase responsible for the chirality of C3 in ACT **1** [15–16]. Since two chiral centers (C3*S* and C15*R*) in the pyran ring of MED **2** are identical to those in ACT **1** (Fig 1A), *med*-ORF12 in the *med* cluster was proposed to determine the chirality of C3 in MED **2** [12, 14].

In the earlier stages of ACT **1** pathway, the bicyclic intermediate **4** could be converted into two shunt products, DMAC (3,8-dihydroxy-1-methylanthraquinone-2-carboxylic acid, **5**) and aloesaponarin II (3,8-dihydroxy-1-methyl-9,10-anthraquinone, **6**) at the absence of ActVI-ORF1(Fig 1B), while ActVI-ORF1 was convincingly verified to convert the bicyclic intermediate **4** into (*S*)-DNPA (4-dihydro-9-hydroxy-1-methyl-10-oxo-3-*H*-naphtho-[2,3-*c*]-pyran-3-(*S*)-acetic acid, **7**), which was the first chiral intermediate in ACT **1** pathway (Fig 1B) [4, 15, 17–18].

Our previous studies revealed that *med*-ORF12 could restore ACT **1** production in the *act*VI-ORF1-deficient mutant strain [12]. In addition to that, a co-expression system composed of 6 *act* earlier genes (KS-, CLP-, ACP-, KR-, ARO- and CYC-encoding genes) together with *med*-ORF12 could result in the production of (*S*)-DNPA **7**, proposing that Med-ORF12 could catalyze a stereospecific ketoreduction at C3 of the bicyclic intermediate **4**, formed by 6 *act* earlier genes [12, 19]. However, there have been no direct evidences to reveal the role of *med*-ORF12 in the biosynthetic pathway of MED **2**.

Hence, in the present study, we performed gene knock out and complementation experiments besides bioinformatics analysis, and proved definitely its involvement in the stereochemical control in MED **2** pathway.

## Materials and Methods

### Strains, plasmids, media and reagents

*Streptomyce coelicolor* CH999 (CH999) is an actinorhodin-cluster-deficient strain, and used as host for heterologous expression of the *med* cluster here [20]. The strain CH999/pIK340 was a medermycin-producing recombinant strain [14], here acting as wild type strain (WT). *Streptomyce sp*. AM-7161 was a medermycin-producing wild type strain [14]. *Escherichia coli* ET12567/pUZ8002 was used for intergeneric conjugation [20]. pIK340 is a plasmid carrying an entire medermycin biosynthetic gene cluster [14]. pRM5 is an expression plasmid containing of 6 *act* earlier genes (KS-, CLP-, ACP-, KR-, ARO- and CYC-encoding genes) [19]. pT7Blue (Novagen) is used as cloning vector, while pYH7, a conditional suicide vector derived from pHZ1358, is used here for knock-out of *med*-ORF12 [21]. pIJ8600 is an inducible vector used for the expression of strepetomyce genes [20].

Media for streptomycete cultivation include YEME, GYM, 2×YT, R4, R5, R5MS (R5 without sugar) and MS media [20, 22]. *E*. *coli* cells were cultivated on LA agar or liquid medium [23]. Recombinant strains derived from *Streptomyces* or *E*. *coli* were selected using ampicillin (100 μg/ml as working concentration), apramycin (50 μg/ml) or thiostrepton (25 μg/ml).

### DNA manipulation and general genetic manipulations

Isolation, restriction enzyme digestion and transformation of DNAs and plasmids were performed according to standard protocols [20, 23]. Plasmids were introduced into the *Streptomyces* strains by intergeneric conjugation between *E*. *coli* and *Streptomyces* hosts [20].

### Bioinformatics analysis

Sequence alignment between Med-ORF12 and its homologies was conducted using the Clustal X program. version 2 [24–25]. The phylogenetic tree was established based on sequence alignment using distance/neighbor joining programs with the Poisson correction distance model in MEGA software package version 4.0 [26]. The interior branch length supports were from 1000 replicates. The role of genes or gene cluster was predicated using online programs of CDD (http://www.ncbi.nlm.nih.gov/Structure/cdd/wrpsb.cgi), Blast (http://blast.ncbi.nlm.nih.gov/Blast.cgi), STRING (version: 9.05) (http://string905.embl.de). and antiSMASH (http://www.secondarymetabolites.org/) [27]. The structural modeling of Med-ORF12 was performed using PyMOL (http://www.pymol.org/) and modeled with NADPH using Autodock (http://mgltools.scripps.edu/).

### Construction of the suicide plasmid for knock-out of *med*-ORF12

**Fig 3:**
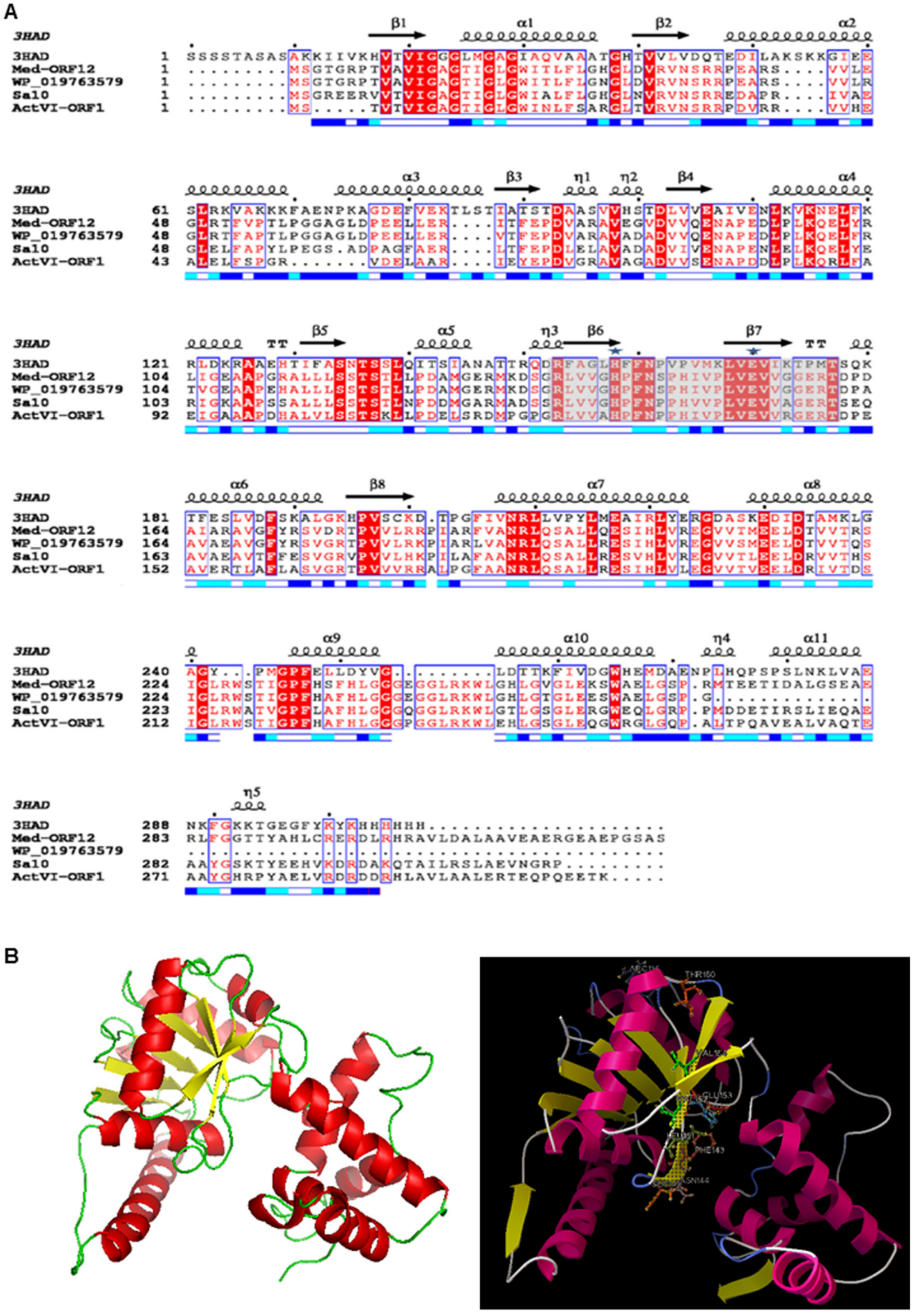
Structure-based sequence alignment between Med-ORF12 and its homologies and the modeled structure of Med-ORF12 . A: The primary sequence of Med-ORF12 is aligned with the ActVI-ORF1, 3HAD, Sa10 and WP_019763579. The sequences are annotated with corresponding secondary structures in 3HAD. Arrows represent β -sheets and helices indicate α-helices. The similar residues are shaded and identical residues are highlighted in black. The key catalytic residues are marked by ☆. The numbers indicating amino acid positions of each entry refer to the nucleotide sequence deposited in the databases. Sequences retrieved from GenBank were aligned using Clustal X. B: Homology model of Med-ORF12 using one chain of 3HAD from human heart as template. Left: The homology modeled structure of Med-ORF12 obtained using PyMOL. β-strands and α-helices are shown in yellow and red, respectively. Right: The NADH molecule is modeled in active sites of Med-ORF12 by the docking program Autodock. The NADPH is shown in cyans sticks, the catalytic dyad (His141 and Glu153) shown in magenta sticks, the residues (Pro142, Phe143, Asn144, Ser229 and Thr230) interacted by hydrogen bonding with NADH shown as sky-blue sticks and the hydrogen bonds shown in blue dots.

For in-frame deletion of *med*-ORF12, firstly, a 1.5 kb fragment (namely “left arm”) containing the upstream region of *med*-ORF12 was amplified using pIK340 as template and a pair of primers (med12-L1/ med12-L2, listed in Table 1). The PCR reaction was performed using KOD-Plus polymerase from TOYOBO and under the conditions composed of 25 recycling reactions as: 1 min at 55°C for denaturation, 45 sec at 65°C for annealing and 1 min at 68°C for extension. Similarly, using another primer pair (med 12-R1/med12-R2, in Table 1), a 1.3 kb downstream fragment (right arm) was amplified (Fig 3A).

**Table 1:** Primers used in this study.

Name	^a^ Sequences (5’-3’)	PCR products
med12-L1	5’-CCCAAGCTTGGTATCGGCACCTCTTC (*Hin*dIII)	1.5 kb DNA located in the upstream of *med*-ORF12
med12-L1	5’-GCTCTAGA **CAT**CGTTTTCG TTCTCCCG (*Xba*I)	
med12-R2	5’-GCTCTAGA **TGA**GGGCGCCGTGCGGTC (*Xba*I)	1.3 kb DNA located in the downstream of *med*-ORF12
med12-R2	5’-GGAATTCAAGCTTGCGGACCAGAACCAG (*Eco*RI-*Hin*dIII)	
med12-qc1	5’-CCGTCCTCCGTGGTGATCTCGAAG	1.7 kb/0.5 kb fragment (wild type/mutant) flanking upstream and downstream of *med*-ORF12
med12-qc2	5’-GTTGACGACGAACTCGGACGCGGC	
med12-A	5’-GGAATTCCAT**ATG**AGCGGAACCGGCCGGCC (*Eco*RI- *Nde*I)	0.975 kb full-length *med*-ORF12
med12-B	5’-CGGGATCC **TCA**CGACGCGCTCCC GGGCT (*Bam*HI)	

Note:

^a^: Sequences underlined in the primers were recognition sites for restriction enzymes indicated in parentheses. Start/stop codons (ATG/TGA) were shown in bold.

After double digestion using *Hin*dIII and *Xba*I (for left arm) and *Xba*I and *Eco*RI (for right arm) respectively, these two PCR products were cloned onto pT7Blue generating two plasmids designated as pHSL121 and pHSL122, respectively. After being sequenced, two arms were linked together on pT7Blue via restriction sites (*Hin*dIII, *Xba*I and *Eco*RI sites, specifically-designed in the primers in Table 1) to give a plasmid as pHSL123, followed by ligating these two linked regions with the suicide vector pYH7. The final plasmid was designated as pHSL124. It was introduced into *E*. *coli* host ET12567/pUZ8002 for subsequent intergeneric conjugation.

### In-frame knock-out of *med*-ORF12

The suicide plasmid pHSL124 was transferred from *E*. *coli* ET12567/(pUZ8002+pHSL124) into the medermycin-producing wild type strain WT via intergeneric conjugation [20]. After selection for apramycin- and thiostrepton- resistance, six conjugants were picked out randomly and verified by PCR with a primer set as med12-qc1/med12-qc2 (Table 1). Next, two rounds of cultivation of conjugant spores on GYM medium without supplementation of either thiostrepton or apramycin were performed for double cross-over between two arms and their homologous regions on the genome of the wild type strain WT. Several proposed *med*-ORF12 deficient mutants (designated as MS) were proved by antibiotic-resistance selection and PCR amplification using two primers (med12-qc1/med12-qc2).

### Construction of the expression plasmid for complementary analysis of *med*-ORF12

The 975 bp *med*-ORF12 gene in a full-length was amplified using a primer set (med12-A/ med12-B, Table 1) and plasmid pIK340 as a template for PCR. The resultant product was cloned onto the *Hin*dIII-*Bam*HI sites of pT7Blue to give a plasmid pHSAY18. After being sequenced, the insert was cleaved out from pT7Blue and ligated with the integrating vector pIJ8600, generating a recombinant plasmid pHSAY19, which was transferred into the *med*-ORF12-deficient mutant strain MS for complement analysis. The obtained transformant strains were proved by PCR using two primers (med12-A/med12-B, Table 1)

### Metabolite analysis of streptomyces strains by LC/MS

To prepare crude extracts from streptomycete cultures, 100 μl of fresh *Streptomyces* spores (1.0x10^6^ spores/μl) were inoculated into 10 ml of TSB liquid medium and grown at 30°C for 48 h. Then, 2 ml of the resultant cultures were added to 100 ml of R5MS medium [22] in a 500-ml Erlenmyer flask. After 5 d incubation on a shaker (200 rpm) at 30°C, supernatants were collected by centrifugation at 6000 rpm for 10 min, followed by extraction with EtoAc [15].

Subsequently, the crude extracts were dissolved in 1 ml of EtoAc and subjected to LC/MS analysis on the system of Agilent 1100 HPLC/Brucker Esquire HCT under the following conditions: column: TSK gel ODS-100S (5 μm, 15.0 cm×4.6 mm, TOSOH); column temperature: 40°C; eluent A: H_2_O + 0.1% HCOOH; eluent B: CH_3_CN + 0.1% HCOOH; gradient elution: 0–35 min (10–95% B), 35–40 min (95% B), 40–45 min (95–10% B); flow rate: 1.0 ml/min; detection wavelength: 254 nm and 434 nm; ionization mode: APCI(-) [14].

### LC/HRMS (High Resolution Mass Spectrum) analysis of two shunt products

Crude extracts from the liquid culture of the *med*-ORF12-deficient strain were subjected to LC/HRMS measurement on the Bruker Compact (Qq-TOF LC-MSMS mass spectrometer system) in a full scan in a negative ionization mode (APCI-). During HPLC analysis, two shunt products were detected at 254 nm and eluted with a gradient as described as above (section: *Metabolite analysis of streptomycete cultures by LC/MS*). During subsequent HRMS analysis, the parent ions with m/z 297.0405 for compound DMAC **5** (C_16_H_9_O_6_^-^ ([M-H]^-^)) and with m/z 253.0506 for compound aloesaponarin II **6** (C_15_H_9_O_4_^-^ ([M-H]^-^)) were monitored and then fragmented.

## Results

### Bioinformatics analysis of Med-ORF12

Conserved domain search suggested that Med-ORF12 is a member of 3-hydroxyacyl-CoA dehydrogenase protein family (3HAD). 3HADs (EC 1.1.1.35) are related with the fatty acid metabolism and reversibly catalyze the β-oxidation of the hydroxyl group of L-3-hydroxyacyl-CoA into a keto group [28](Fig 2C).

Blast search showed that many members in 3HAD family possess a quite high identities to Med-ORF12, and are widely distributed in many bacteria, most of which are *Actinobacteria*, such as *Streptomyces*, *Frankia*, *Nocardiopsis*, *Kibdelosporangium* and *Amycolatopsis*, while *Streptomyces* make up a largest portion among these bacteria.

Many homologues in the *streptmyces* species are involved in the secondary metabolism in *Streptomyces*: for example, ActVI-ORF1 from *S*. *coelicolor* [16] was proved functionally to be an stereospecific ketoreductase involving in actinorhodin biosynthesis [15–16]. Sa10 annotated as a putative 3-hydroxyacyl-CoA dehydrogenase gene (having an identity of 74% to Med-ORF12) was encoded by the indigoidine/auricin hybrid cluster from *Streptomyces aureofaciens* [29–30]. It is noteworthy that a hypothetical protein (WP_019763579) from *Streptomyces sp*. Wigar10 has a highest identity (91%) with Med-ORF12. Using antiSMASH program online, we found that it was located within a secondary metabolite gene cluster, which shows extremely high resemblance to the gene cluster for medermycin biosynthesis. So, we deduced WP_019763579 to be involved in the biosynthesis of an unknown natural product [31].

It is well known that ketoreductases (KR) are widely distributed in the pathways of aromatic polyketide natural products. For example, the keto-reduction by KR in the nascent polyketide chains is a common and critical step for the formation of aromatic polyketide skeletal core in the earlier stages [6, 19]. The stereochemistry at C3 of MED **2** via a stereospecific keto-reduction might be controlled by also a ketoreductase, either Med-ORF6 or Med-ORF12 (only two possible candidates encoded by *med* cluster). So, we established a phylogenetic tree using proposed ketoreductases in BIQ pathways and several closer homologies using MEGA 4.09. Fig 2B showed that these homologies involving microbial secondary metabolism could be divided into three groups, though PSI-Blast revealed that they were divided into two families: Members in Group I and II belong to the family of short-chain alcohol dehydrogenases (SDR-family) [32] while members in Group III belong to that of L-3-hydroxyacyl-CoA dehydrogenase (3HAD-family) (Fig 2B).

Even we used more homologues from *streptomyces* species and a non-streptomyces species for phylogenetic analysis and found they could be categorized into group III (Figure A in S1 File). Blast search for the downstream and upstream genes of these homologues in their genomes supported that most of them might be related to the biosynthesis of secondary metabolites, though which structures remain unknown [33–36].

Several closer homologies in Group III and Med-ORF12 were multiply aligned with Clustal X, showing that some conserved sites commonly exist in these enzymes, such as His141/Glu153 for Med-ORF12 and His129/Glu141 for ActVI-ORF1. Secondary structures analysis revealed that eight β-sheets and eleven helixes are aligned in a similar mode in all examples (Fig 3A).

Subsequently, we performed structural modeling of Med-ORF12 using 3HAD from human heart as reference protein template (Med-ORF12 has 27% identity to 3HAD) [28], revealing that Med-ORF12 shares a similar structure to 3HAD (Fig 3B). Similar to the subunit of 3HAD, the first domain of Med-ORF12 is composed of alpha/beta dinucleotide folds and an unusual helix-turn-helix motif extending from the central beta-sheet, forming a Rossman fold, which should be for NADPH-binding. The second domain at C-terminal is composed of primarily alpha-helical, and might mediate subunit dimerization. Fig 3B also showed that these conserved sites are located in the proposed catalytic center of Med-ORF12.

### Inactivation of Med-ORF12

**Fig 4:**
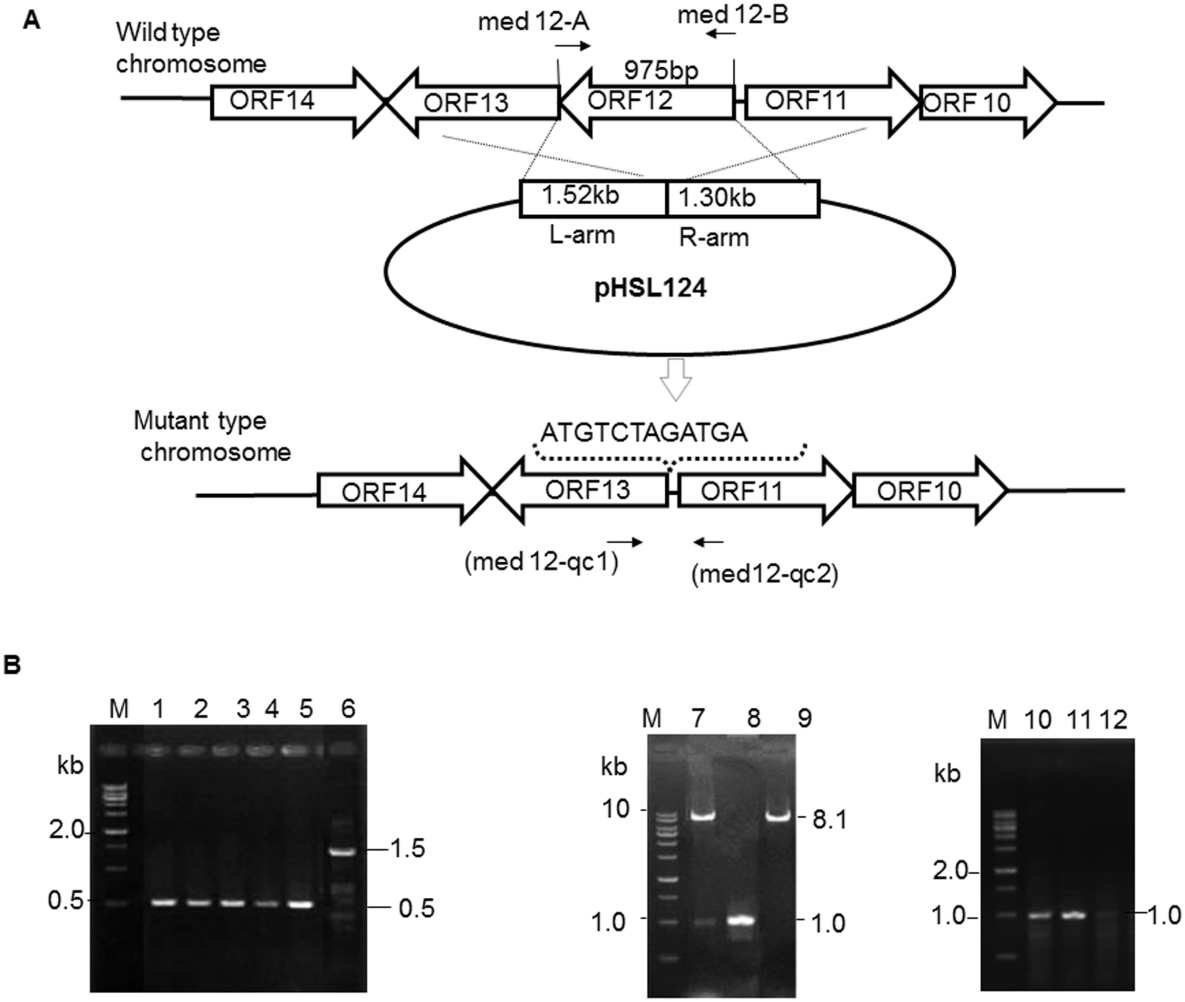
Inactivation and complementation of *med*-ORF12. A: Schematic representation of knock-out *med*-ORF12. A 2.8 kb genomic fragment is composed of left arm (L-arm) and right arm (R-arm) referring to the upstream and downstream regions flanking *med*-ORF12 respectively. These two DNA regions are linked together and then inserted into a suicide vector pYH7, giving a suicide plasmid pHSL124. After double- crossover between chromosome of the wild type strain and homologous regions on pHSL124, *med*-ORF12-deficient mutant strain was acquired. On the chromosome of the mutant, an artificially-designed sequence (**ATG** TCTAGA **TGA**) containing *Xba*I site in underline and start codon/stop codon in bold to replace in-frame *m*ed-ORF12 in the full-length. B: PCR confirmation of *med*-ORF12-deficient mutant strain (MS) and complementary strain (CS). The amplification using wild-type strain genomic DNA (lane 6) as template and a primer set (med12-qc1/ med12-qc1, showed in arrow in A) produced a 1 488 bp PCR product, while the genomic DNA of the *med*-ORF12- deficient mutant strains (lane 1–5) gave a 513 bp band as expected. The 975 bp genomic fragment of *med*-ORF12 was amplified by PCR (lane 8) and inserted into an integrative vector pIJ8600 (8.1 kb, lane 9: digestion with *Nde*I and *Bam*HI), generating an expression plasmid pHSAYT19 (lane 7: double digestion with *Nde*I and *Bam*HI). Genomic DNA isolated from the complementary strains (lane 10) and a primer set (med 12-A/med 12-B shown in arrow in A) were used for PCR amplification, giving a 975 bp band indicating the presence of *med*-ORF12, as same as for wild type strain genomic DNA as template (lane 11). On the contrary, the *med*-ORF12-deficient mutant strain (lane 12) could not produce an expected a 975 bp PCR product. M is 1 kb ladder as DNA marker.

To verify the role of Med-ORF12 in the biosynthetic pathway of MED **2**, we conducted knock-out experiments: a conditional suicide plasmid pHSL124 was constructed using pYH7 as vector (Fig 4A). pYH7 is an automatically-replicating vector under the selection of thiostrepton, but acts as a suicide plasmid without thiostrepton selection [21]. An artificially-designed sequence (**ATG**TCTAGA**TGA**) was designed to replace in-frame the full gene of *med*-ORF12. Subsequently, pHSL124 was introduced into the wild type strain WT.

We picked out several apramycin- or thiostrepton-sensitive colonies and confirmed them to be *med*-ORF12-deficient strains (designated as MS) (Fig 4B) by PCR amplification.

**Fig 5:**
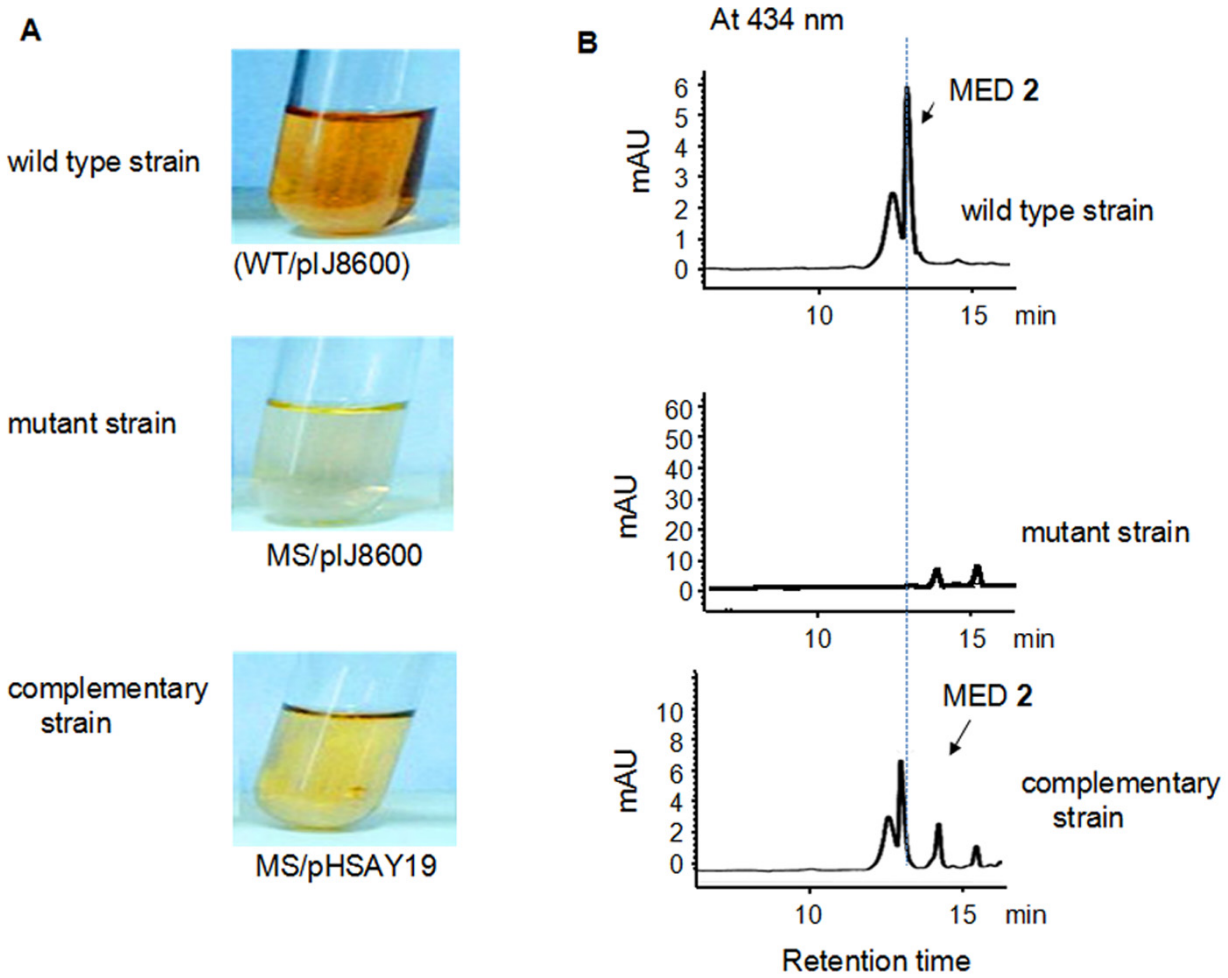
Metabolite analysis of *Streptomyces* strains. A: Comparison of the pigmentation in liquid cultures between medermycin-producing wild type strain (WT) and its derived strains. Red-brown pigmentation acts as indicator for MED **2** production. The mutant strain (MS) was a *med*-ORF12 deficient strain. The complementary strain was obtained by transforming the expression plasmid pHSAY19, derived from pIJ8600, into *med*-ORF12 deficient mutant strain. Expression of *med*-ORF12 on pHSAY19 in the complementary strain was induced by thiostrepton (12.5 mg/ml). The wild type and mutant strains were transformed with the vector pIJ8600. B: HPLC spectra of metabolites in wild type, mutant and complementary strains, indicated as UV absorption at 434 nm of crude extracts of *Streptomyces* strains in A. In a contrast to wild type strain (WT/pIJ8600), the mutant strain (MS/pIJ8600) could not produce MED **2** due to the deficiency of *med*-ORF12. The reintroduction of *med*-ORF12 could restore the production of MED **2** in the complementary strain in a comparable yield to that in the wild type strain.

The supernatants of mutant strains cultivating on R5MS liquid medium were analyzed by HPLC for MED **2** production, using the wild type strain as positive control. Red-brownish pigmentation in the MED-producing strain acted as indicator for MED **2** production [14]. Fig 5A showed *med*-ORF12 deficiency caused pigmentation disappearance in the supernatants of the mutant strain grown on liquid medium, in a sharp contrast to the wild type strain, implying the disappearance of MED **2** production. HPLC measurements (Fig 5B and Figure B in S1 File) demonstrated clearly that MED **2** production was abolished completely in *med*-ORF12-deficient mutant strain.

### Complementary analysis of Med-ORF12

In order to characterize further the role of *med*-ORF12, we performed complementary experiments to investigate if the expression of *med*-ORF12 could restore *med*-ORF12-deficiency. An expression plasmid pHSAY19 was constructed derived from an integrative vector pIJ8600, which was designed for expression of *med*-ORF12 under the control of the inducible thiostrepton promoter, tipAp [20]. After introducing it into the mutant strain MS, we verified the integration of *med*-ORF12 in the chromosome of the host MS by PCR amplification (Fig 4B).

After 5 d expression induced by thiostrepton supplemented into the liquid culture of the complementary strain MS/pHSAY19, the supernatant showed obvious restoration in pigmentation, on the contrary to the mutant strain MS/pIJ8600 (Fig 5A). HPLC measurement suggested that complementary strain could produce MED **2** with a comparable yield as in the wild type strain (Fig 5B).

### Accumulation of two shunt products at the absence of *med*-ORF12

**Fig 6:**
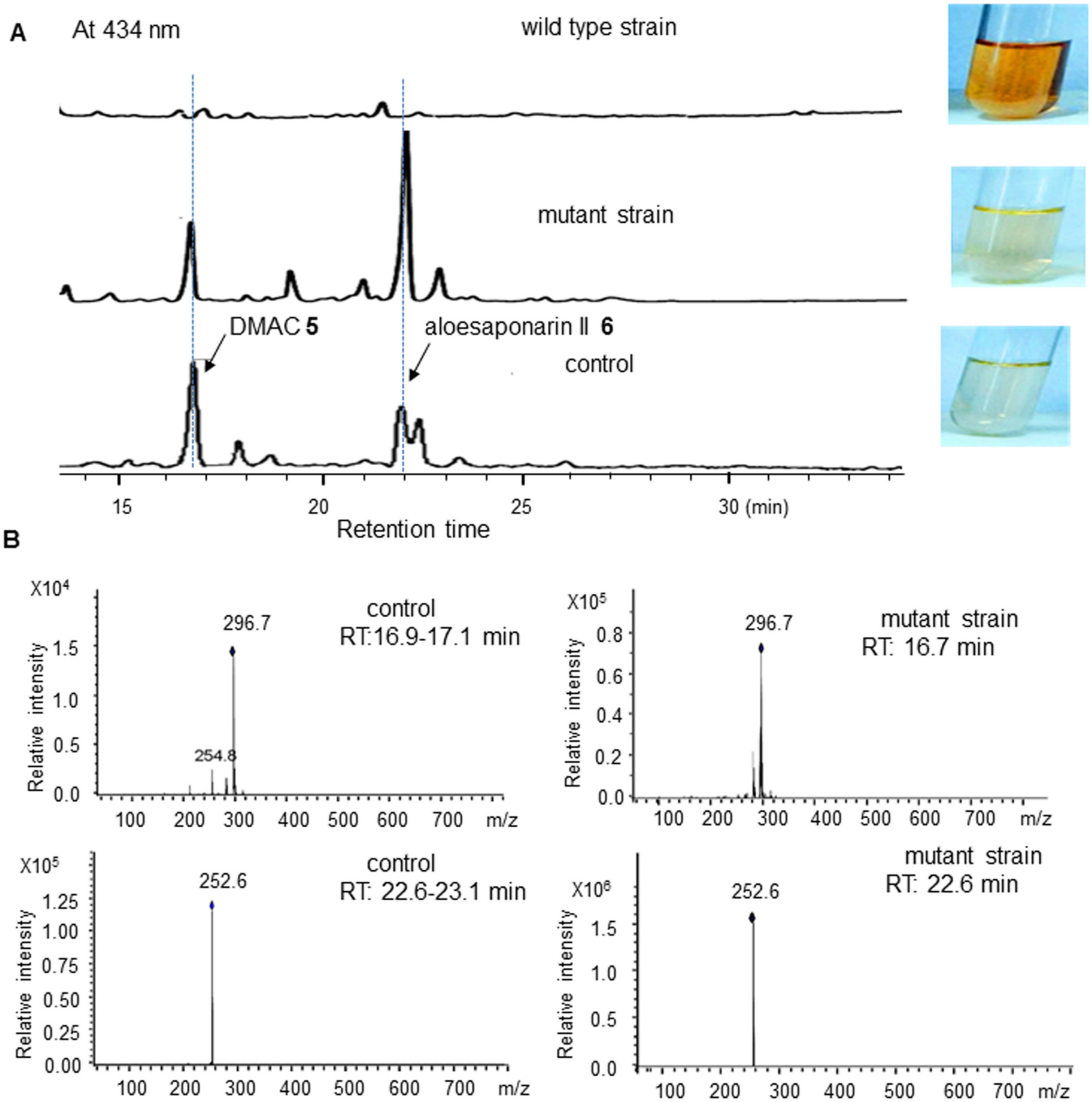
Detection of two shunt products in the mutant strain. A: HPLC spectra of metabolites by the wild type strain (WT/pIJ8600), mutant strain (MS/pIJ8600) and control strain (CH999/pRM5), indicated as UV absorption at 434 nm. Control: CH999/pRM5 was a recombinant strain of *S*. *coeliololar* able to accumulate two shunt products DMAC **5** (Mr: 298, 16.7 min) and aloesaponarin II **6** (Mr. 254, 22.6 min), due to the presence of 6 genes on pRM5, encoding KS, CLF, ACP, KR, ARO and CYC (Fig 1B) from the earlier stages of ACT **1** pathway. These 6 genes control the formation of the bicyclic intermediate **4**. Then the bicyclic intermediate was spontaneously converted into two shunt products in CH999/pRM5 (Fig 1B). B: Mass spectra of peaks for authentic DMAC **5** and aloesaponarin II **6** by CH999/pRM5 and for the compounds at 16.9 min and 22.6 min by the *med*-ORF12- deficient mutant strain.

In a sharp contrast to the wild type strain, LC/MS measurement (Fig 6A) showed that two compounds indicated as two peaks at 16.7 and 22.6 min were accumulated significantly in the *med*-ORF12 deficient mutant strain (Figure B in S1 File also showed several other new peaks in a lower amount between 15–25 min besides these two major ones, in a contrast to the wild type strain).

CH999/pRM5 was a producer of DMAC **5** and aloesaponarin II **6**, two shunt products in ACT **1** pathway (Fig 1B). Using CH999/pRM5 as a positive control, it was observed that two compounds by the *med*-ORF12-defieicent mutant strain showed identical retention time and mass spectra to these shunt products by CH999/pRM5 (Fig 6). HRMS analysis of these two shunt products further suggested this mutant strain could accumulate DMAC **5** and aloesaponarin II **6** (Figure C and Figure D in S1 File).

## Discussion

### Essential role of Med-ORF12 in the biosynthesis of medermycin

Stereochemical control is a common and important tailoring modification during the biosynthetic pathways of many polyketide antibiotics, including aromatic polyketide antibiotics daunorubicin and ACT **1** (Fig 1A), and well-known macrolide antibiotic avemectin (Fig 1) [37]. Understanding the stereochemistry in these compounds is of highly chemical, biological and pharmacological importance for generating hybrid natural products with improved pharmacological profiles via metabolic engineering in a combinatorial fashion.

MED **2** possesses 7 stereospecific centers (Fig 1). In the present study, our investigation focused on the conFig uration at C3 center of its pyran ring. we characterized functionally a ketoreductase-encoding gene *med*-ORF12 located in *med* cluster by knock-out and complementary experiments, and found that its deficiency caused disappearance of MED **2**. Meanwhile, introduction of *med*-ORF12 into this mutant strain could restore the production of MED **2**. Our data here offered the direct evidences to reveal its critical role in the biosynthesis of MED **2** for the first time.

### Involvement of *med*-ORF12 in the stereochemical control

In the present study, we also observed two shunt products were accumulated at the absence of *med*-ORF12 (Fig 6). As shown in Fig 1B, at the presence of 6 *act* genes for earlier stages of ACT **1** pathway, the bicyclic intermediate **4** was produced, but further entered into a shunt pathway if *act*VI-ORF1 is absent [15]. So, *act*VI-ORF1-deficient strain could accumulate two shunt products DMAC **5** and aloesaponarin II **6**. The recombinant strain *S*. *coelicolor* CH999/pRM5 was also able to produce these two shunt products because pRM5 carried 6 genes encoding KS, ACP, CYC, KR, ARO and CYC from ACT pathway (Fig 1B), though CH999 is an entire-ACT-cluster-deficient mutant from *S*. *coelicolor* [15,19].

Our previous studies proved that Med-ORF12 could convert the bicyclic intermediate **4** into (*S*)-DNPA **7** with a conFig uration (3*S*) at C3, when working together with these 6 *act* early biosynthetic genes encoding KS, ACP, CYC, KR, ARO and CYC [12]. This suggested that Med-ORF12 could recognize the bicyclic intermediate **4** as substrate and play a similar role to ActVI-ORF1. Hence, it is predicted that two shunt products DMAC **5** and aloesaponarin II **6** could be accumulated in the *med*-ORF12-deficient strain. LCMS and HRMS measurements in the present study proved such a speculation, using CH999/pRM5 as a control (Fig 6 and Figure C and Figure D in S1 File).

All these evidences supported that Med-ORF12 could play an essential role in the MED **2** pathway, as a ketoreductase stereospecific for the formation of the chiral center at C3 of MED **2**.

### Three groups of ketoreductases in the biosynthetic pathways of polyketides

Ketoreductases belong to the family of oxidoreductases and widely distributed in the pathways of aromatic polyketide natural products. Here, our phylogenetic analysis revealed that these ketoreductases from *Streptomyces* strains and even from non- *Streptomyces* strain could be divided into three groups and PSI-Blast search further demonstrated that group I and II belong to SDR-family and group III belongs to 3HAD-family (Fig 3B and Figure A in S1 File)

Therefore, the closer evolutionary distance between Group I and II allowed us to deduce that both of them might originate from a common ancestor, though they undergo different evolutionary routes to acquire different function: Group I represents region-specific ketoreductases (KR), exampled by ActIII for keto-reduction at C-9 of ACT **1**, determining the precise position for the folding of a 16-membered polyketide chain before aromazation and cyclization [3–5]. All the conserved and characteristic core motifs and amino-acid residues of the domains are present in all the members in group I.

Among the members in Group III and II, only ActVI-ORF1, Gra-ORF6 and Med-ORF12 have been characterized functionally.

Though the members in Group II (SDR family) have quite low identities to those in Group III (3HAD family), previous evidences suggested that both of them might be involved in the enantiomerical control in the pathways of polyketides, as stereo-specific ketoreductases:

1. Gra-ORF6 in group II and ActVI-ORF1 in Group III were revealed to function as stereospecific ketoreductases in the pathways of GRA **3** (for 3*R* conFig uration) and ACT **1** (for 3*S* conFig uration) respectively (Fig 1A) [15–16]. Consistently, 3HAD catalyzes the fatty acid metabolism as stereo-specific ketoreductase (Fig 2C).
2. AveF and DnrU in group II were proposed to be ketoreductases for stereochemical control at C-5 in avemectin and at C13 in daunorubicin respectively [13–14], though no direct experimental evidences support the functions of them.

We also found the highest homologue WP_019763579 (identity: 91%) might be involved in the biosynthesis of a medermycin-like compound (though it was annotated as a hypothetical protein in GenBank), because antiSMASH analysis supported that WP_019763579 is located within a *med*-like gene cluster in the genome of *Streptomyces sp*. Wigar10 [31]. Additionally, based on the phylogenetic tree in Figure A in S1 File, we proposed more proteins classified into group III might have a similar role to Med-ORF12 and ActVI-ORF1.

Examples in these two groups might perform different mechanisms: previous biochemical analysis *in vitro* and *in vivo* suggested Gra-ORF6 and ActVI-ORF1 control completely opposite chirality in GRA **3** and ACT **1** pathways respectively, though they could recognize an identical substrate (the common bicyclic intermediate **4**) [15–16]. It is noteworthy that AveF and DnrU also control different opposite chirality in avemectin (C5*R*) and daunorubicin (C13*S*) pathways, but both of them are present in a same group. Hence, the mechanisms of members in Group II and III need to be further clarified.

### Sequence comparison between 3HAD and members in group III

Med-ORF12 belongs to the family of 3HAD. 3HAD could catalyze a reversible reaction between L-3-hydroxyacyl-CoA and 3-oxoacyl-CoA and mainly exist in mammals (Fig 2C). Some members of 3HAD family are also found in many microorganisms, including the examples in Group III. It is noteworthy that 3-oxoacyl-CoA, the substrate for 3HAD in mammals, showed a partially similar structure to the reactive portion of the bicyclic intermediate **4**, the common substrate for ActVI-ORF1 and Med-ORF12 (Figs 1B and 2).

Molecular modeling studies and biochemical observations of 3HAD from human heart elucidated that two sites (His158 and Glu170) constituted a catalytic dyad located in the active site region [28]. As the first example characterized to be involved in microbial secondary metabolism in Group III, ActVI-ORF1 also contains two conserved residues (His129 and Glu141),which were proved to be critical for the reduction of the C-3 keto group of the bicyclic intermediate **4** [16]. In the present study, we found these two sites are highly conserved in all the members of Group III, further indicating their importance for the reducing activity of these enzymes.

Further, similar to ActVI-ORF1 and 3HAD, we found these two residues are located in the active site region in the modeled structure of Med-ORF12 (Fig 3B), as the second example characterized to be involved in microbial secondary metabolism in Group III. This implied that Med-ORF12 controls the chiral conformation at C3 of MED **2** via a similar mechanism to ActVI-ORF1 and 3HAD: In the catalytic dyad composed of Glu153 and His141, the carboxyl group of Glu153 is responsible for the activation of the proton in His141 side chain, probably via nucleophillic attack. Then the activated proton of the imidazolyl ring of His141 mediated the electron transfer from C-3 keto-group of the substrate. Accordingly, the pro-S hydride from NAD(P)H was transferred to the C-3 of the bicyclic intermediate **4**, leading to the formation of *S* conFig uration.

We have established prokaryotic expression and purification system of Med-ORF12 [38] and hope to perform enzymatic assay *in vitro* in next steps to investigate its mechanism.

In a conclusion, stereochemical control in BIQ pathways is one of most important tailoring modification. In the present study, functional characterization by gene knockout, gene complementary, LC/MS and HRMS analysis elucidated convincingly that Med-ORF12 is essential for the production of MED **2** as a stereospecific ketoreductase and reduces the keto-group at C3 during MED **2** pathway to form a specific conFig uration (3S). These data supported further that MED **2** and ACT **1** share parts of tailoring modification steps besides their earlier stages, and will help us understand better the stereochemistry in BIQ pathways as an important step towards metabolic engineering to generate a novel pharmacophore.

## Supporting Information

S1 File Bioinformatics analysis of Med-ORF12 and HRMS spectra of two shunt products. **Figure A**: Phylogenetic tree established using more homologues of Med-ORF12. These entries include proteins involved in biosynthesis of secondary metabolites: Med-ORF12 and Med-ORF6 (for MED **2** in *Streptomyces sp* AM-7161), ActVI-ORF1 and ActIII (for ACT **1** in *S*. *coelicolor* A3(2)), DauB (for aklaviketone in *Streptomyces* sp.), Gra-ORF5 and Gra-ORF6 (for GRA **3** in *Streptomyces violaceoruber*, AveF (for avermectin in *Streptomyces avermitilis*), DnrU(for daunorubicin in *Streptomyces peucetius*), Sa10 (for indigoidine/auricin in *Streptomyces aureofaciens*), 3HAD (3HAD_B) from human heart and a hypothetical protein WP_019763579 (encoded by a cluster highly in *Streptomyces sp* Wigar10, homologous to medermycin cluster). Additionally, several highly homologous proteins proposed to encode hydroxylacyl-CoA dehydrogenases were also analyzed: WP_033529872 from *Streptomyces galbus* (identity to Med-ORF12: 80%), WP_030735501from *Streptomyces* sp. (66%), WP_043385010 from *Streptomyces mutabilis* (66%), WP_030080416 from *Streptomyces sp*. (65%), WP_042193116 from *Kibdelosporangium sp*. (65%) and WP_030666394 from *Streptomyces cellulosae* (64%). The bar indicated the evolutionary distance. The numbers on branch nodes were percentages of 1000 sets of bootstrap supports. All homologies are divided into two families (3HAD: 3-hydroxyacyl-CoA dehydrogenase protein family; SDR: short-chain alcohol dehydrogenases family) and further into three groups. **Figure B**: Comparison of metabolite production between the wild type strain WT/pIJ8600 (A) and *med*-ORF12-deficient strain MS/pIJ8600 (B and C). Crude extracts isolated from the wild type and mutant strains respectively were subjected to HPLC analysis, indicated as UV absorption at 434 nm. The Y axils of B and C (both for mutant strain) were adjusted into different scales for better comparison with the wild type strain. In a contrast to the wild type strain (WT/pIJ8600, A), the mutant strain (MS/pIJ8600) could not produce MED **2** due to the deficiency of *med*-ORF12, but could produce many intermediates or shunt products (12–25 min), which were not present in the wild type strain. Among of them, two major components (deduced to be DMAC **5** and aloesaponarin II **6**) were further analyzed in next experiments. **Figure C**: HRMS analysis of aloesaponarin II **6** from the control strain CH999/pRM5 (A and C) and *med*-ORF12-deficient strain MS/pIJ8600 (B and D). The parent ions were detected in A and B (HR-MS, A and B) using atmospheric pressure chemical ionization (APCI-), and then further fragmented (HR-MS_2_, C and D). **Figure D**: HRMS analysis of DMAC **5** from the control strain CH999/pRM5 (A) and *med*-ORF12-deficient strain MS/pIJ8600 (B). The parent ions were detected in A and B (HR-MS, APCI-).
(DOCX)

## References

[1] Challis GL. Genome mining for natural product discovery. J Med Chem. 2008; 51: 2618–2628. 10.1021/jm700948z 18393407

[2] Zhang H, Wang H, Wang Y, Cui H, Xie Z, Pu Y, et al Genomic sequence-based discovery of novel angucyclinone antibiotics from marine *Streptomyces sp*. W007. FEMS Microbiol Lett. 2012; 332(2): 105–112. 10.1111/j.1574-6968.2012.02582.x 22536997

[3] Ichinose K, Taguchi T, Ebizuka Y & Hopwood DA. Biosynthetic gene clusters of benzoisochromanequinone antibiotics in Streptomyces spp.-identification of genes involved in post-PKS tailoring steps. Actinomycetologica. 1998; 12: 99–109.

[4] Metsä-Ketelä M, Oja T, Taguchi T, Okamoto S & Ichinose K. Biosynthesis of pyranonaphthoquinone polyketides reveals diverse strategies for enzymatic carbon-carbon bond formation. Curr Opin Chem Biol. 2013; 17(4): 562–570. 10.1016/j.cbpa.2013.06.032 23886982

[5] Donner CD. The divergent asymmetric synthesis of kalafungin, 5-epi-frenolicin B and related pyranonaphthoquinone antibiotics. Tetrahedron. 2013; 69 (2013): 377–386.

[6] Hopwood DA. Genetic contributions to understanding polyketide synthases. Chem Rev. 1997; 97: 2465–2497. 11851466 10.1021/cr960034i

[7] Rix U, Fischer C, Remsing LL & Rohr J. Modification of post-PKS tailoring steps through combinatorial biosynthesis. Nat Prod Rep. 2002; 19: 542–580. 12430723 10.1039/b103920m

[8] Takano S, Hasuda K, Ito A, Koide Y, Ishii F, Haneda I, et al A new antibiotic, medermycin. J Antibiot. 1976; 29: 765–768. 956059 10.7164/antibiotics.29.765

[9] Salaski EJ, Krishnamurthy G, Ding WD, Yu K, Insaf SS, Eid C, et al Pyranonaphthoquinone lactones: a new class of AKT selective kinase inhibitors alkylates a regulatory loop cysteine. J Med Chem. 2009; 52(8): 2181–2184. 10.1021/jm900075g 19309081

[10] Toral-Barza L, Zhang WG, Huang X, McDonald LA, Salaski EJ, Barbieri LR, et al Discovery of lactoquinomycin and related pyranonaphthoquinones as potent and allosteric inhibitors of AKT/PKB: mechanistic involvement of AKT catalytic activation loop cysteines. Mol Cancer Ther. 2007; 6(11): 3028–3038. 17989320 10.1158/1535-7163.MCT-07-0211

[11] Madduri K & Hutchinson CR. Functional characterization and transcriptional analysis of a gene cluster governing early and late steps in daunorubicin biosynthesis in Streptomyces peucetius. J Bacteriol. 1995; 177(13): 3879–3884. 7601857 10.1128/jb.177.13.3879-3884.1995 PMC177111

[12] Li A, Itoh T, Taguchi T, Xiang T, Ebizuka Y & Ichinose K. Functional studies on a ketoreductase gene from Streptomyces sp. AM-7161 to control the stereochemistry in medermycin biosynthesis. Bioorg Med Chem. 2005; 13(24): 6856–6863. 16169737 10.1016/j.bmc.2005.07.060

[13] Ichinose K, Bedford DJ, Tornus D, Bechthold A, Bibb MJ, Revill WP, et al The granaticin biosynthetic gene cluster of Streptomyces violaceoruber Tü22: sequence analysis and expression in a heterologous host. Chem Biol. 1998; 5(11): 647–659. 9831526 10.1016/s1074-5521(98)90292-7

[14] Ichinose K, Ozawa M, Itou K, Kunieda K & Ebizuka Y. Cloning, sequencing and heterologous expression of the medermycin biosynthetic gene cluster of Streptomyces sp. AM-7161: towards comparative analysis of the benzoisochromanequinone gene clusters. Microbiology. 2003; 149(7): 1633–1645. 12855716 10.1099/mic.0.26310-0

[15] Taguchi T, Itou K., Ebizuka Y, Malpartida F, Hopwood DA, Surti CM. Chemical characterisation of disruptants of the Streptomyces coelicolor A3(2) actVI genes involved in actinorhodin biosynthesis. J Antibiot (Tokyo). 2000; 53(2): 144–152. 10805574 10.7164/antibiotics.53.144

[16] Taguchi T, Kunieda K, Takeda-Shitaka M, Takaya D, Kawano N, Kimberley MR, et al Remarkably different structures and reaction mechanisms of ketoreductases for the opposite stereochemical control in the biosynthesis of BIQ antibiotics. Bioorg Med Chem. 2004; 12(22): 5917–5927. 15498668 10.1016/j.bmc.2004.08.026

[17] Taguchi T, Yabe M, Odaki H, Shinozaki M, Metsä-Ketelä M, Arai T, et al Biosynthetic conclusions from the functional dissection of oxygenases for biosynthesis of actinorhodin and related streptomyces antibiotics. Chem & Biol. 2013; 20: 510–520. 23601640 10.1016/j.chembiol.2013.03.007

[18] Itoh T, Taguchi T, Kinberley MR, Booker-Milburn KI, Stephenson GR, Ebizuka Y, et al Actinorhodin biosynthesis: structural requirements for post-PKS tailoring intermediates revealed by functional analysis of ActVI-ORF1 reductase. Biochemistry. 2007; 46(27): 8181–8188. 17579485 10.1021/bi700190p

[19] McDaniel R, Ebert-Khosla S, Hopwood DA & Khosla C. Engineered biosynthesis of novel polyketides. Science. 1993; 262: 1546–1550. 8248802 10.1126/science.8248802

[20] Kieser T, Bibb MJ, Buttner MJ, Chater KF & Hopwood DA. Practical Streptomyces Genetics. John Innes Foundation, Norwich, UK, 2000

[21] Sun Y, He X, Liang J, Zhou X & Deng Z. Analysis of functions in plasmid pHZ1358 influencing its genetic and structural stability in Streptomyces lividans 1326. Appl Microbiol Biotechno. 2009; 82(2):303–310. 10.1007/s00253-008-1793-7 19066884

[22] Okamoto S, Taguchi T, Ochi K, Ichinose K. Biosynthesis of actinorhodin and related antibiotics: discovery of alternative routes for quinone formation encoded in the act gene cluster. Chem Biol. 2009; 16(2): 226–236. 10.1016/j.chembiol.2009.01.015 19246012

[23] Sambrook J & Russell D. Molecular Cloning, A Laboratory Manual. 3rd ed Cold Spring Harbor Laboratory Press, New York 2001.

[24] Larkin MA, Blackshields G, Brown NP, Chenna R, McGettigan PA, McWilliam H, et al Clustal W and Clustal X version 2.0. Bioinformatics. 2007; 23: 2947–2948. 17846036 10.1093/bioinformatics/btm404

[25] Thompson JD, Gibson TJ, Plewniak F, Jeanmougin F & Higgins DG. The ClustalX windows interface: flexible strategies for multiple sequence alignment aided by quality analysis tools. Nucl Acids Res. 1997; 25: 4876–4882. 9396791 10.1093/nar/25.24.4876 PMC147148

[26] Tamura K, Dudley J, Nei M & Kumar S. MEGA4: Molecular evolutionary genetics analysis (MEGA) software version 4.0. Mol Biol Evol. 2007; 24: 1596–1599. 17488738 10.1093/molbev/msm092

[27] Blin K, Medema MH, Kazempour D, Fischbach MA, Breitling R, Takano E, et al antiSMASH 2.0—a versatile platform for genome mining of secondary metabolite producers. Nucleic Acids Res. 2013; 41(Web Server issue):W204–212. 10.1093/nar/gkt449 23737449 PMC3692088

[28] Barycki JJ, O'Brien LK, Bratt JM, Zhang R, Sanishvili R, Strauss AW, et al Biochemical characterization and crystal structure determination of human heart short chain L-3-hydroxyacyl-CoA dehydrogenase provide insights into catalytic mechanism. Biochemistry. 1999; 38: 5786–5798. 10231530 10.1021/bi9829027

[29] Novakova R, Bistakova J, Homerova D, Rezuchova B & Kormanec J. Cloning and characterization of a polyketide synthase gene cluster involved in biosynthesis of a proposed angucycline-like polyketide auricin in *Streptomyces aureofaciens* CCM 3239. Gene. 2002; 297(1–2): 197–208. 12384301 10.1016/s0378-1119(02)00889-2

[30] Novakova R, Odnogova Z, Kutas P, Feckova L & Kormanec J. Identification and characterization of an indigoidine-like gene for a blue pigment biosynthesis in Streptomyces aureofaciens CCM 3239. Folia Microbiol (Praha). 2010; 55(2): 119–125. 20490753 10.1007/s12223-010-0018-5

[31] Klassen JL, Adams SM, Bramhacharya S, Giles SS, Goodwin LA, Woyke T. et al Draft genome sequence of Streptomyces sp. strain Wigar10, isolated from a surface-sterilized garlic bulb. J Bacteriol. 2011; 193(24): 6999–7000. 10.1128/JB.06257-11 22123757 PMC3232846

[32] Persson B & Kallberg Y. Classification and nomenclature of the superfamily of short-chain dehydrogenases/reductases (SDRs). Chem-Biol Interact. 2013; 202(1–3):111–115. 10.1016/j.cbi.2012.11.009 23200746

[33] Kim HJ, Karki S, Kwon SY, Park SH, Nahm BH, Kim YK. A single module type I polyketide synthase directs de novo macrolactone biogenesis during galbonolide biosynthesis in *Streptomyces galbus* . J Biol Chem. 2014; 289(50):34557–34568. 10.1074/jbc.M114.602334 25336658 PMC4263863

[34] Ogasawara Y, Torrez-Martinez N, Aragon AD, Yackley BJ, Weber JA, Sundararajan A. High-quality draft genome sequence of Actinobacterium Kibdelosporangium sp. MJ126-NF4, producer of Type II polyketide azicemicins, using Illumina and PacBio technologies. Genome Announ. 2015; 3(2). 10.1128/genomeA.00114-15 PMC4384478 25838474

[35] Fehr T, Kuhn M, Loosli HR, Ponelle M, Boelsterli JJ & Walkinshaw MD. 2-Epimutalomycin and 28-epimutalomycin, two new polyether antibiotics from *Streptomyces mutabilis*. derivatization of mutalomycin and the structure elucidation of two minor metabolites. J Antibiot (Tokyo). 1989; 42(6):897–890. 2737949 10.7164/antibiotics.42.897

[36] Li Z, Rawlings BJ, Harrison PH, Vederas JC. Production of new polyene antibiotics by *Streptomyces cellulosae* after addition of ethyl (Z)-16-phenylhexadec-9-enoate. J Antibiot (Tokyo). 1989; 42(4):577–584 2722673 10.7164/antibiotics.42.577

[37] Omura S, Ikeda H, Ishikawa J, Hanamoto A, Takahashi C, Shinose M, et al Genome sequence of an industrial microorganism Streptomyces avermitilis: deducing the ability of producing secondary metabolites. Proc Natl Acad Sci, USA. 2001; 98 (21): 12215–12220. 11572948 10.1073/pnas.211433198 PMC59794

[38] Sun R, Liu M, Gong C, Wang W, Zeng A & Li A. Polyclonal antiserum preparation and expression detection of *med*-ORF12 encoding a stereochemical ketoreductase possibly involved in medermycin biosynthesis. Acta Microbiol Sinica. 2012; 52(1): 60–68. 22489461

