# Supplementary material for: Functional Characterization of a Ketoreductase-Encoding Gene med-ORF12 Involved in the Formation of a Stereospecific Pyran Ring during the Biosynthesis of an Antitumor Antibiotic Medermycin
Source: PLoS One. 2015 Jul 10;10(7):e0132431. doi: 10.1371/journal.pone.0132431 (PMC4498746; doi:10.1371/journal.pone.0132431)
Supplement: S1 File — Figure A: Phylogenetic tree established using more homologues of Med-ORF12. These entries include proteins involved in biosynthesis of secondary metabolites: Med-ORF12 and Med-ORF6 (for MED 2 in Streptomyces sp AM-7161), ActVI-ORF1 and ActIII (for ACT 1 in S. coelicolor A3(2)), DauB (for aklaviketone in Streptomyces sp.), Gra-ORF5 and Gra-ORF6 (for GRA 3 in Streptomyces violaceoruber, AveF (for avermectin in Streptomyces avermitilis), DnrU(for daunorubicin in Streptomyces peucetius), Sa10 (for indigoidine/auricin in Streptomyces aureofaciens), 3HAD (3HAD_B) from human heart and a hypothetical protein WP_019763579 (encoded by a cluster highly in Streptomyces sp Wigar10, homologous to medermycin cluster). Additionally, several highly homologous proteins proposed to encode hydroxylacyl-CoA dehydrogenases were also analyzed: WP_033529872 from Streptomyces galbus (identity to Med-ORF12: 80%), WP_030735501from Streptomyces sp. (66%), WP_043385010 from Streptomyces mutabilis (66%), WP_030080416 from Streptomyces sp. (65%), WP_042193116 from Kibdelosporangium sp. (65%) and WP_030666394 from Streptomyces cellulosae (64%). The bar indicated the evolutionary distance. The numbers on branch nodes were percentages of 1000 sets of bootstrap supports. All homologies are divided into two families (3HAD: 3-hydroxyacyl-CoA dehydrogenase protein family; SDR: short-chain alcohol dehydrogenases family) and further into three groups. Figure B: Comparison of metabolite production between the wild type strain WT/pIJ8600 (A) and med-ORF12-deficient strain MS/pIJ8600 (B and C). Crude extracts isolated from the wild type and mutant strains respectively were subjected to HPLC analysis, indicated as UV absorption at 434 nm. The Y axils of B and C (both for mutant strain) were adjusted into different scales for better comparison with the wild type strain. In a contrast to the wild type strain (WT/pIJ8600, A), the mutant strain (MS/pIJ8600) could not produce MED 2 due to the deficiency of med-ORF [file pone.0132431.s001.docx]

**Supporting Information**


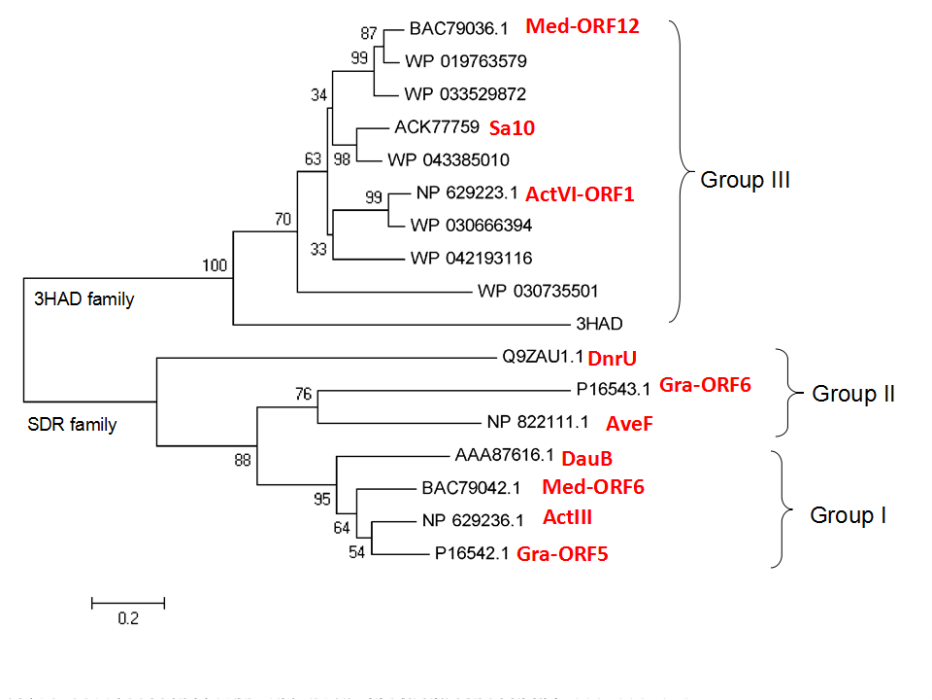


# Figure A Phylogenetic tree established using more homologues of Med-ORF12. These entries include proteins involved in biosynthesis of secondary metabolites: Med-ORF12 and Med-ORF6 (for MED 2 in *Streptomyces sp* AM-7161), ActVI-ORF1 and ActIII (for ACT 1 in *S. coelicolor* A3(2)), DauB (for aklaviketone in *Streptomyces* sp.), Gra-ORF5 and Gra-ORF6 (for GRA 3 in *Streptomyces violaceoruber*, AveF (for avermectin in *Streptomyces avermitilis*), DnrU(for daunorubicin in *Streptomyces peucetius*), Sa10 (for indigoidine/auricin in *Streptomyces aureofaciens*), 3HAD (3HAD_B) from human heart and a hypothetical protein WP_019763579 (encoded by a cluster highly in *Streptomyces sp* Wigar10, homologous to medermycin cluster). Additionally, several highly homologous proteins proposed to encode hydroxylacyl-CoA dehydrogenases were also analyzed: WP_033529872 from *Streptomyces galbus* (identity to Med-ORF12: 80%), WP_030735501from *Streptomyces* sp. (66%), WP_043385010 from *Streptomyces mutabilis* (66%), WP_030080416 from *Streptomyces sp*. (65%), WP_042193116 from *Kibdelosporangium sp.* (65%) and WP_030666394 from *Streptomyces cellulosae* (64%). These strains are producers of natural products. The bar indicated the evolutionary distance. The numbers on branch nodes were percentages of 1000 sets of bootstrap supports. All homologies are divided into two families (3HAD: 3-hydroxyacyl-CoA dehydrogenase protein family; SDR: short-chain alcohol dehydrogenases family) and further into three groups.

#
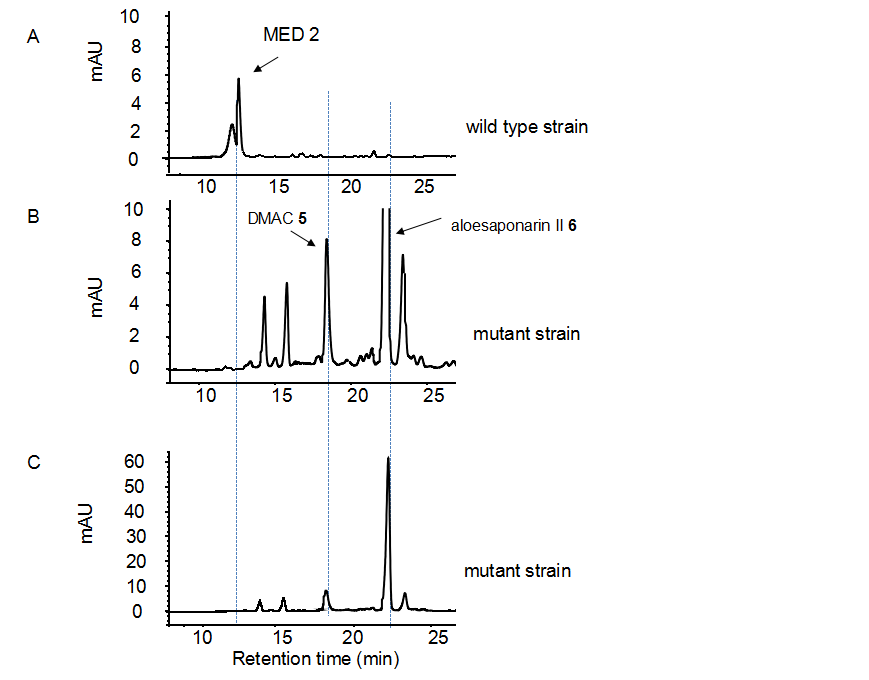


**Figure B** Comparison of metabolite production between the wild type strain WT/pIJ8600 (A) and *med*-ORF12-deficient strain MS/pIJ8600 (B and C). Crude extracts isolated from the wild type and mutant strains respectively were subjected to HPLC analysis, indicated as UV absorption at 434 nm. The Y axils of B and C (both for mutant strain) were adjusted into different scales for better comparison with the wild type strain. In a contrast to the wild type strain (WT/pIJ8600, A), the mutant strain (MS/pIJ8600) could not produce MED 2 due to the deficiency of *med*-ORF12, but could produce many intermediates or shunt products (12-25 min), which were not present in the wild type strain. Among of them, two major components (deduced to be DMAC 5 and aloesaponarin II 6) were further analyzed in next experiments.


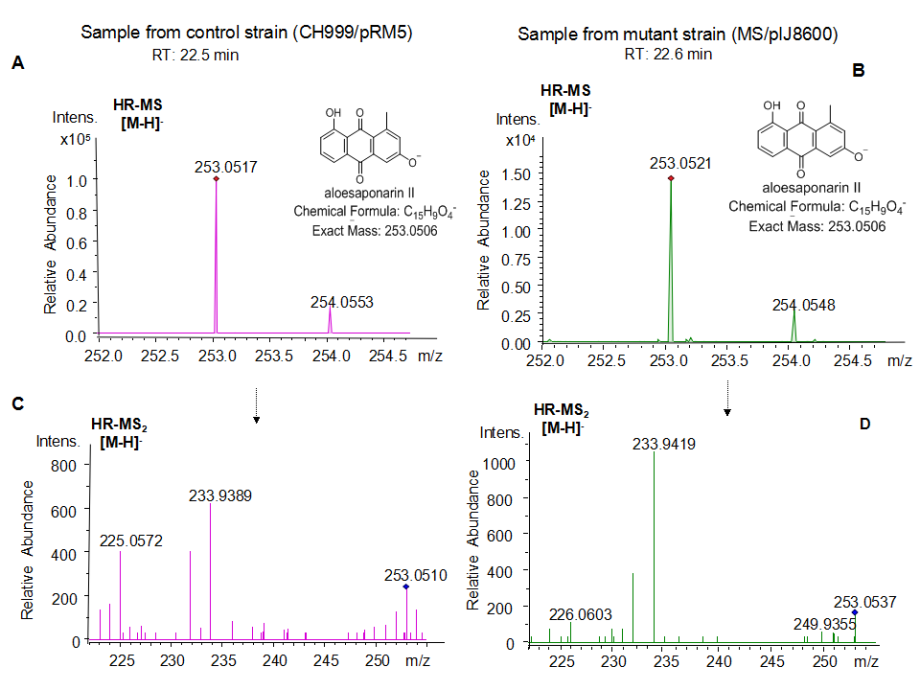


**Figure C** HRMS analysis of aloesaponarin II 6 from the control strain CH999/pRM5 (A and C) and *med*-ORF12-deficient strain MS/pIJ8600 (B and D). The parent ions were detected in A and B (HR-MS, A and B) using atmospheric pressure chemical ionization (APCI-), and then further fragmented (HR-MS_2_, C and D).


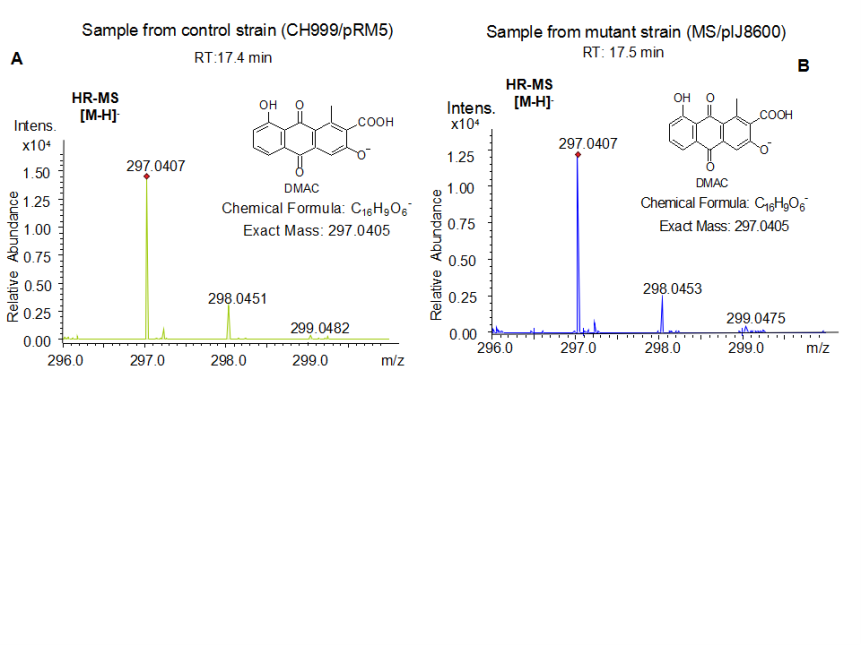


**Figure D** HRMS analysis of DMAC 5 from the control strain CH999/pRM5 (A) and *med*-ORF12-deficient strain MS/pIJ8600 (B). The parent ions were detected in A and B (HR-MS, APCI-).
